# Induction of apoptosis by casticin in cervical cancer cells through reactive oxygen species-mediated mitochondrial signaling pathways

**DOI:** 10.3892/or.2022.8448

**Published:** 2022-11-18

**Authors:** Dan Chen, Jianguo Cao, Li Tian, Fei Liu, Xifeng Sheng

Oncol Rep 26: 1287–1294, 2011; DOI: 10.3892/or.2011.1367

Following the publication of the above paper, it was drawn to the Editors' attention by a concerned reader that certain of the lanes shown in the DNA agarose gel electrophoresis experiment in Figs. 2D bore some striking similarities; furthermore, there were unexpectedly similar-looking bands included within the gel slices for the western blotting experiments portrayed in Fig. 3B and C. The Editor of *Oncology Reports* independently received a request from the authors that this paper be retracted, and in view of the uncertainties regarding some of the data shown in Figs. 2 and 3, the Editor agrees with the authors’ request that their paper be retracted. The Editor apologizes to the readership for any inconvenience caused.

